# Spatial mapping of single cells in the *Drosophila* embryo from transcriptomic data based on topological consistency

**DOI:** 10.12688/f1000research.24163.2

**Published:** 2021-02-09

**Authors:** Maryam Zand, Jianhua Ruan

**Affiliations:** 1 Computer science, University of Texas at San Antonio, San Antonio, Texas, 78249, USA

**Keywords:** Single cell RNA-seq, spatial mapping, feature selection, particle swarm intelligence, nearest neighbor

## Abstract

The advancement in single-cell RNA sequencing technologies allow us to obtain transcriptome at single cell resolution. However, the original spatial context of cells, a crucial knowledge for understanding cellular and tissue-level functions, is often lost during sequencing. To address this issue, the DREAM Single Cell Transcriptomics Challenge launched a community-wide effort to seek computational solutions for spatial mapping of single cells in tissues using single-cell RNAseq (scRNA-seq) data and a reference atlas obtained from in situ hybridization data. As a top-performing team in this competition, we approach this problem in three steps. The first step involves identifying a set of most informative genes based on the consistency between gene expression similarity and cell proximity. For this step, we propose two different approaches, i.e., an unsupervised approach that does not utilize the gold standard location of the cells provided by the challenge organizers, and a supervised approach that relies on the gold standard locations. In the second step, a Particle Swarm Optimization algorithm is used to optimize the weights of different genes in order to maximize matches between the predicted locations and the gold standard locations. Finally, the information embedded in the cell topology is used to improve the predicted cell-location scores by weighted averaging of scores from neighboring locations. Evaluation results based on DREAM scores show that our method accurately predicts the location of single cells, and the predictions lead to successful recovery of the spatial expression patterns for most of landmark genes. In addition, investigating the selected genes demonstrates that most predictive genes are cluster specific, and stable across our supervised and unsupervised gene selection frameworks. Overall, the promising results obtained by our methods in DREAM challenge demonstrated that topological consistency is a useful concept in identifying marker genes and constructing predictive models for spatial mapping of single cells.

## Introduction

Single cell RNA sequencing (scRNA-seq) is a cost-efficient, high throughput technology that has dramatically enhanced our understanding of developmental biology such as cell type identification, regulatory network inference, and cell trajectories ^1– 8^. Despite many breakthroughs in biological sciences made possible by this technology, it yet suffers from the drawback that native cell location in e.g. embryo or complex tissue is often lost, except for in a few experimental methodologies which are either expensive, require highly specialized tools, or are not as widely applicable as standard scRNA-seq protocols ^9– 12^. Given the substantial benefit offered through cell location recovery, such as obtaining a basic understanding of tissue function and disease pathology ^13, 14^, the cell spatial reconstruction was specifically addressed in recent Single Cell Transcriptome DREAM challenge as a community-wide effort.

Many promising computational approaches dealing with the spatial reconstitution problem are centered around the main idea that an *in situ* atlas of a set of landmark gene’s expressions is used as a guideline to be combined with scRNA-seq profiles of individually measured cell ^15, 16^. For instance, Seurat ^15^ first imputes the noisy scRNAseq data then predicts the cell locations by comparing the scRNAseq gene expression pattern to its binary expression level measured by *in situ* data. This step is done through a mixture model. Finally, original cell location is retrieved by evaluating a posterior probability function constructed for cell-bin pairs. DistMap ^16^ was a successful method for spatial reconstruction (of *Drosophila* embryo) with near single cell resolution, much higher compared to that of Seurat (3039 bins versus 128 bins). It predicts top candidate positions for a given cell by calculating the Mathews Correlation Coefficients (MCC) of binarized landmark gene expressions for every cell-bin combination. While DistMap was to some extent successful in dealing with the cell spatial mapping problem, it was limited to binarized data rather than continuous, utilized simplistic MCC analysis, and more importantly it treats each single cell independently whereas it might be more beneficial to account for collective interrelationships between cells. To more extensively explore the space of better predictive strategies, DREAM challenge aimed to exploit the atlas provided by DistMap with the hope of resolving spatial reconstruction by using incrementally fewer landmark genes (i.e. 60,40,20). Achieving this goal will help with eliminating the need for *a priori* reference atlas, which is expensive and time-consuming to obtain, in the future transcriptomic studies.

In this work, we proposed a top-performing method (evaluated based on three distinct scoring criteria defined by DREAM challenge) which allows us to predict the cell location consistently as accurate as DistMap while requiring fewer number of landmark genes. The details of our method and evaluation metrics are provided later in the text.

## Methods

### Overview of the proposed method

The general overview of our method is such that in the first step we investigate both supervised and unsupervised feature selection methods by defining two biologically rational metrics optimizing the consistency between gene expression similarity and cell proximity. In the unsupervised version we do not use the predicted cell locations given in 16 to obtain the set of most informative genes (e.g. 60,40,20), thus avoiding overfitting. On the other hand, the supervised version uses the cell locations given by DistMap as a reference. In the next, to predict the final cell locations, we use a PSO algorithm to assign proper weights to genes based on fitness functions defined by gene expression patterns. This reflects the intuition that different landmark genes are expected to demonstrate different potential in guiding us toward the proper embryo reconstruction. Finally, we use the information embedded in the cell topology to adjust the associated cell-location score with the hope to improve the predictions.

### Datasets and pre-processing steps

To reconstruct *Drosophila* embryo from single cells, we need reference dataset ( *in situ*), spatial coordinates, and scRNA-seq data, the details of which along with the preprosseing steps are given in the following.

**Reference database** The reference database (denoted as *W*) provides the *in situ* expression values as a *W*_3039×84_ matrix where rows and columns correspond to bin locations and marker genes, respectively. The original data comes from Berkeley Drosophila Transcription Network Project (BDTNP) and in here we used the binarized format as explained in 16.

**Spatial coordinates** The spatial coordinate information from one half of *Drosophila* embryo (denoted as *L*) is an *L*_3039×3_ matrix where the columns are *x*, *y*, and *z* coordinates of 3039 rows of bins.

**Single cell RNA sequencing** The scRNA-seq data (denoted as *Y*) gives the gene expression values as a *Y*_1297×8924_ matrix where rows and columns are single cells and genes, respectively. In here we followed the normalization process as implemented by 16. Briefly, the raw data was first normalized with respect to the total number of unique molecular identifiers (UMI) for each cell, followed by a pseudo count addition and a log transformation. The binarization process was implemented such that the quantile was varied in order to obtain the minimum mean squared root error between the gene correlation matrix of binarized atlas and binarized scRNA-seq.

**Gold standard cell locations:** For each of the 1297 cells, the Mathews Correlation Coefficients (MCC) is calculated at each of the 3039 location bins between the binarized 84 RNAseq expression values for the 84 driver genes and the binarized *in situ* expression values for the same 84 genes. The location bin with the maximum value of MCC score is defined as the gold standard location for each cell.

### Finding most informative genes

In this study, our first goal is to identify a subset of genes whose expression patterns are predictive of cell locations. We have proposed two different feature selection methods (supervised and unsupervised) to select informative genes. In the supervised method, our metric was defined based on true cell locations (gold standard). To prevent overfitting we applied a 10 fold cross validation. On the other hand, we designed an unsupervised method based on the intuition that the current locations obtained by matching the normalized and binarized scRNA-seq expression patterns with the *in situ* expression patterns are not necessarily the true locations of these cells. These two methods are discussed in detail in the following sections.

### Unsupervised gene selection

As we believe the current locations obtained by matching the normalized and binarized scRNA-seq expression patterns with the *in situ* expression patterns are not necessarily the true locations of these cells, we decided to take an unsupervised feature selection approach, which does not depend on the current locations of the cells to be predicted, and therefore avoid overfitting.

The key rationale in our unsupervised feature selection method is that if a set of genes can be used as predictors of cell locations, then the cells showing similar expression patterns of these genes must be geometrically close to each other. Therefore, we defined two complementary metrics to quantitatively measure the proximity of cells with similar expression patterns for different gene subsets, and developed a greedy algorithm to search for a gene subset with the optimal (minimal) score combining the two metrics.

***Metrics to measure the power of gene signatures as location predictors***. The first metric relies solely on the *in situ* gene expression patterns in the 3039 location bins, and is calculated as follows: given a set of genes *G* as features, the pairwise Pearson Correlation Coefficient (PCC) is computed between the *in situ* expression data for every pair of the 3039 location bins; the top-10 locations with the highest PCC is then identified for each location bin; the metric $M_{1}^{G}$ is defined as the average Euclidean distance between each location bin and its top-10 most similar location bins:

$$M_{1}^{G}=\frac{\sum_{i=1}^{n}\sum_{j\in L_{ki}^{G}}D_{ij}}{k\times n},\;(1)$$

where $L_{\text{ki}}^{G}$ is the set of *k* most similar bins for location *i* based on the *in situ* expression pattern of a gene signature *G*, *k* is fixed at 10 in this work, and *n* =3039 is the total number of location bins. *D _ij_* is the Euclidean distance between the geometric coordinates of location *i* and location *j*. In this work, *k* is set to 10 because the evaluation of the prediction results is based on 10 best locations for each single cell. Also, based on the number of location bins ( *n*), we believe 10 is a reasonable choice for the number of nearest neighbors.

The second metric uses information from both the *in situ* expression data and the scRNA-seq expression data, and is calculated as follows. Given a set of genes *G* as features, the pairwise PCC is computed between the scRNA-seq expression pattern of each of the 1297 cells and the *in situ* expression pattern of each of the 3039 location bins; then for each of the 1297 cells, the top-10 location bins with the highest PCC is identified; the metric $M_{2}^{G}$ is defined as the average Euclidean distance between the geometric coordinates of the location bin most similar to cell *c* and the geometric coordinates of the top-10 most similar location bins (including the most similar location):

$$M_{2}^{G}=\frac{\sum_{c=1}^{m}\sum_{j\in S_{kc}^{G}}D_{l_{c}^{G}j}}{k\times m},\;(2)$$

where $S_{\text{kc}}^{G}$ is the set of top- *k* locations whose *in situ* expression patterns are most similar to the scRNA-seq expression pattern of cell *c* based on gene signature *G*, *k* is fixed at 10 in this work, and *m* =1297 is the total number of cells whose locations are to be predicted. $l_{c}^{G}$ is the location bin where the expression pattern of gene signature *G* is most similar to cell *c*.

Note that the currently known most possible location of each cell *c*, $l_{c}^{*}$ which is predicted using all 84 genes with uniform weights, are not used in either *M*_1_ and *M*_2_; therefore, the gene selection process is not biased towards identifying genes to match the original locations predicted by the 84 genes. Rather, the metric provides an intrinsic measurement of the power of any subset of genes as location predictors, independent of the locations predicted with the 84 genes. In fact, the quality of the 84 genes as predictors can also be measured using these two metrics, and compared to any other gene sets; it is possible that a subset of the 84 genes can receive higher scores in these two metrics than the original 84 genes. In contrast, using a supervised feature selection method, where the “true” location is defined using all 84 genes, any subset of genes will be necessarily inferior to the complete set of 84 genes.

***Step-wise backward elimination feature selection algorithm***. We used a standard backward elimination algorithm to identify a subset of genes *G* with the minimal sum of $M_{1}^{G}\;\text{and}\;M_{2}^{G}$. Briefly, starting with a set of *q* genes, we computed $M_{1}^{G}\;\text{and}\;M_{2}^{G}$ for all possible subsets of *q* − 1 genes by removing one gene at a time from the set. The subset with the minimal $M_{1}^{G}+M_{2}^{G}$ is then recorded as the best subset of size *q* − 1. This procedure is then repeated until a desired number of genes is reached. As this algorithm is a greedy approach, it does not guarantee to find the optimal solution. We have also attempted to combine backward elimination with forward selection, which only improved the solution slightly. Due to the excessive running time required, we opted to use the simple algorithm described above while leaving additional improvement as future work.

### Supervised gene selection

While in the unsupervised approach metrics *M*1 and *M*2 were optimized, in the supervised version a single metric *N* was defined as explained below. This metric, which relies on both the scRNA-seq gene expression patterns in the 1297 cells and the gold standard location of each cell, is calculated as follows: given a set of genes *G* as features, the pairwise PCC is computed between the scRNA-seq expression data for every pair of the 1297 cells; the top-10 cells with the highest PCC is then identified for each cell; the metric *N ^G^* is defined as the average Euclidean distance between the gold standard geometric coordinates of each cell and its top-10 most similar cells:

$$N^{G}=\frac{\sum_{c=1}^{m}\sum_{j\in T_{kc}^{G}}D_{l_{c}^{*}l_{j}^{*}}}{k\times m},\;(3)$$

where $T_{\text{kc}}^{G}$ is the set of top- *k* cells whose scRNA-seq expression patterns are most similar to the scRNA-seq expression pattern of cell *c* based on gene signature *G*, *k* is fixed at 10 in this work, and *m* =1297 is the total number of cells whose locations are to be predicted. $l_{c}^{*}$ is the “gold standard” cell location for cell *c*, which is predicted using all 84 genes.

### Supervised learning to find optimal gene weights

It is intuitive to assume that the contribution of genes in determining cell locations are not equal. Therefore, we look for a way to learn how to assign proper weight to each selected gene for more accurate prediction of cell locations. To this end, we chose a supervised learning approach, using the cell locations predicted by the highest MCCs with the 84 signature genes as “gold standard” locations. To avoid overfitting, we performed 10-fold cross-validation: gene weights were determined using the scRNA-seq data of 90% of cells; these weights are then used to predict the locations of the remaining 10% of the cells not used in training. The splitting of the data is saved, for reproducibility of the results.

The basic idea of the PSO algorithm is as follows. We created a set of agents, each of which is initiated with a gene weight vector *w _i_* of size | *G*|× 1. Each weight vector is evaluated by how closely the weighted gene expression pattern can be used to predict the cell locations when compared to the “gold standard” locations obtained with the 84 genes:

$$M_{3}^{w,G}=\frac{\sum_{c=1}^{m}\sum_{j\in S_{kc}^{w,G}}D_{l_{c}^{*}j}}{k\times m},\;(4)$$

where $S_{kc}^{w,G}$ is the set of top- *k* location bins whose *in situ* expression patterns are most similar to the weighted expression pattern of cell *c* based on a given gene signature set *G*. The similarity is measured by PCC here. *k* is fixed at 10, and *m* is the total number of cells in the training set. $l_{c}^{*}$ is the “gold standard” cell location for cell *c*, which is predicted using all 84 genes with uniform weights.

During the search, each agent keeps track of a personal best weight vector *P best _i_*, and the global best solution from all agents is denoted *Gbest*. At each iteration, the weight vector of each agent is updated by the differences between the current weight and the personal best and global best weight vectors:

$$w_{i}=w_{i}+\alpha \times r_{1}\circ \left(Pbest_{i}-w_{i}\right)+\beta \times r_{2}\circ \left(Gbest-w_{i}\right),$$

where *α* and *β* are constants to control the granularity of the search and speed of convergence. We choose *α* = *β* = 0.2 with 200 agents and the maximum number of iterations is 40. The operator ∘ denotes entry-wise vector multiplication. *r*_1_ and *r*_2_ are vectors of random numbers uniformly distributed between 0 and 1, generated independently for each agent at each iteration. *α* and *β* are acceleration coefficients, also referred to as trust parameters. *α* expresses how much confidence a particle has in itself, while *β* expresses how much confidence a particle has in its neighboring particles. Particles draw their strength from their cooperative nature and are most effective when *α* and *β* coexist in a good balance. Most applications use *α*= *β*. Low values for *α* and *β* result in smooth particle trajectories, while high values cause more acceleration, with abrupt movement towards or past good regions. In this study we used *α*= *β*=0.2, with 200 agents and the maximum number of iterations set to 40. These parameters were manually tuned by observing the values of the fitness function to reach desired search granularity and speed of convergence. The running time of PSO algorithm is about 15 hours when running on a system with 16 GB of memory. However, the running time could depend on different optimization settings such as parameter *α* and *β*.

### Neighbor-weighted cell location prediction

The location prediction for each single cell relies on the (weighted) similarity between the expression pattern of selected signature genes in the cell and every location bin. It is important to note that the expression patterns in neighboring cells should be similar in general, and therefore the overall prediction should take the expression of nearby location bins into consideration. Intuitively, if the globally highest scoring location is far away from locations with slightly lower but comparable scores, the confidence score for the highest-scoring location should be reduced; on the other hand, a locally highest-scoring location close to other high-scoring locations should be upweighted. Therefore, to make the final prediction for a given cell, we adjusted the prediction score based on the prediction scores from neighbor locations.

Formally, let *C* = ( *c _ij_*) _*n*× *m*_ be the bin-cell association matrix, where *c _ij_* is the PCC between the (weighted) scRNA-seq data and the *in situ* hybridization data for every pair of cells and locations. *n* = 3039 is the number of candidate location bins, and *m* is the number of cells in the test set, Let *D* = ( *d _ij_*) _*n×n*_ be the Euclidean distance matrix between the geometric coordinates of every pair of location bins. We define an affinity matrix *A* = ( *a _ij_*) _*n×n*_ such that $a_{ij}=e^{-\frac{d_{ij}}{d*}}$, where d* is a parameter to control how many neighbor locations can impact the final prediction score. A smaller d* value means fewer neighbor locations to be considered. To have a robust measure of how geometrically close two location bins can be, we first measure the distance between each location and its nearest location, and then computed the median of these shortest distances as d*. As a result, most *a _ij_*s’ are much smaller than *e* ^−1^, and only a limited number of neighbor locations with very high scores can impact the final prediction score for each cell.

The final prediction score matrix *P* = ( *p _ij_*) _*n*× *m*_ is calculated by *P* = *A × C*. Since *a _ii_* = 1 and *a _ij_* ≤ 1 for all *j* ≠ *i*, it is easy to see that

$$P_{ij}=\sum_{k=1}^{n}a_{ik}c_{kj}=c_{ij}+\sum_{k\neq j}a_{ik}c_{kj}\cdot$$

Therefore, the final prediction score for a cell *i* to be at a particular location *j* is the weighted sum of the similarity scores between the expression pattern of cell *i* and all locations, where the weight is an exponentially decreasing function of the geometric distance from location *j*.

From the final predicted bin-cell association matrix, we reported the 10 locations with the highest scores for each cell as the most likely positions in embryo.

### How proposed method applied to different subchallenges

During the challenge period, each team was given a limited number of attempts to test the success of their proposed approach(es) - evaluation results and ranking for all teams were shown in a leaderboard, with no details of the evaluation metrics. It was made clear that different methods could be used for different sub-challenges. Our final results for subchallenge 1 were obtained with both PSO and neighbor weighting. For subchallenge 2, we were not able to perform PSO due to a lack of time. On the other hand, it was also our observation that PSO only resulted in modest improvement with almost no impact to our ranking based on feedback from previous rounds. Therefore, the 40 genes obtained from gene selection in subchallenge 2 were utilized with uniform weights. In subchallenge 3, genes were weighted with the PSO procedure, but we did not perform neighbor weighting. The rationale is that, as subchallenge 3 used substantially fewer genes, the quality of the location prediction may be relatively low and therefore using gene expression information from the *predicted* neighbors may actually degrade the final prediction.

### Post-challenge phase

In this phase to evaluate the robustness and soundness of the method, a 10 fold CV scenario was performed to obtain 10 different sets of informative genes using a subset of cells. To compare the similarity of the selected genes, Jaccard similarity was defined as follows:

$$\;J\left(A,B\right)=\frac{\left|A\cap B\right|}{\left|A\cup B\right|}\;(5)$$

where *A* and *B* are two sets of informative genes and *J*( *A*, *B*) measures the ratio of the number of common genes and the total number of genes presented in two sets. In addition, the expected Jaccard similarity was computed as follows:

$$E\left(J\right)=\sum_{k=0}^{m}\frac{k}{2m-k}\frac{\left(\begin{array}{c} m\\ k \end{array}\right)\left(\begin{array}{c} n\text{−}m\\ m\text{−}k \end{array}\right)}{\left(\begin{array}{c} n\\ m \end{array}\right)}\;(6)$$

where, *n* is the total number of genes, here 84, and *m* is the number of genes in our selected gene set, 60,40, and 20 for subchallenge 1,2, and 3, respectively.

### DREAM consortium evaluation metrics

Our method was designated as a top-performing method among 34 participating teams. To evaluate and rank the teams, the challenge organizers had defined three scoring metrics s1, s2, and s3, which were not disclosed to participants at the time of submission. The details of each metric are available in 17 and are quite complex. Here, we briefly explain each scoring metric and the general intuition behind them.

The first metric *s*_1_ computes the weighted average of the Mathew Correlation Coefficient (MCC) between the *in situ* profile of the ground truth cell location (as predicted by DistMap) and the *in situ* profile of the most probable prediction location for that cell ^17^.

$$s_{1}=\sum_{c=1}^{N}\frac{p_{K}(c,A)}{\sum_{i=1}^{N}p_{K}(i,A)}MCC(f_{A(c,1,K)},\;f_{{}_{c}})$$

where *N* is the total number of cells with predicted locations, *A*( *c*, *i*, *K*) represents the predicted *i−*th most probable location for cell *c* using *K* genes, _c_ the ground truth location bin for cell *c*, and *f*_*_c_*_ the *in situ* expression profile at _c_ for the *K* selected genes. The weights are calculated as $p_{k}(c,A)=\frac{d_{84}(c,A)}{d_{k}(c,A)},$ where *d _k_*( *c*, *A*) is the average euclidean distance between the geometric coordinates of the ground truth location of cell *c* and the top-10 locations predicted using *k* genes. *d*_84_( *c*, *A*) is the value of *d _k_*( *c*, *A*) using *k* = 84.

The second metric, *s*_2_ only considers how the averaged location prediction of the 10 most probable predictions using 60, 40, and 20 genes is compared to that of the one predicted by using all the 84 genes ^17^. As is evident, this metric does not include either of the accuracy of the *in situ* expression profile prediction and the closeness of *in situ* and scRNA-seq data.

$$s_{2}=\frac{1}{N}\sum_{c=1}^{N}p_{k}(c,A)$$

Finally, *s*_3_ accounts for how the scRNA-seq expression of 60,40, and 20 genes of the best predicated locations is closely approximating that of the *in situ* expression patterns ^17^.

$$s_{3}=\sum_{s=1}^{K}\frac{MCC(t_{cs},\;f_{{}_{c}s})\forall c}{\sum_{i=1}^{K}MCC(t_{ci},\;f_{{}_{i}r})\forall c}MCC(t_{cs},\;f_{A(c,1,K)s})\forall c$$

where *t _cs_* represents the binarized expression value of gene *s* in cell *c*, and ∀ *c* denotes that MCC is calculated cell wise for each gene.

### Software

The method proposed here is written in Matlab 2018b and the source code is available from GitHub ^18^

It does not utilize or rely on any specific Matlab toolbox. Therefore by following the clear detailed formulation provided in manuscript this method can readily be implemented in any open-access software.

## Results and discussion

### Performance evaluation

Table 1 shows the results of our supervised and unsupervised methods on the three subchallenges, evaluated by the three metrics (s1, s2, and s3) proposed by the DREAM challenge organizers. The details of these metrics are discussed in Method Section “DREAM consortium evaluation metrics”. A more detailed analysis of the results and comparison with other top-performing algorithms are presented in 17, and is not repeated here. To obtain some additional insights of our algorithms’ performance, we present here the results of some variations of our proposed methods. Both our supervised and unsupervised methods have two important components, (1) gene selection, and (2) neighbor-weighted cell location prediction, integral to selecting a set of most informative genes and locating cells based on the information buried in cell neighborhood network topology. To understand the importance of these two components, we designed a set of baseline studies incorporating four experiments. In these experiments, the gene selection strategy was replaced by either selecting genes randomly, or selecting genes expressed in the most number of cells (high degree genes). The neighbor-based reweighting component was also removed in two of these experiments.

The subchallenge scores corresponding to our method (supervised and unsupervised) along with these four baseline studies are listed in Table 1 under the group A and group B, respectively. The method with highest score (s1, s2, s3) in each of the three subchallenges is shown in boldface. It can be seen that the supervised and unsupervised methods (group A) achieved comparable results, and significantly outperformed the baseline approaches (group B) on average, for all three subchallenges. For subchallenge 3, which is the most difficult task, both of our methods significantly outperformed the baseline approaches in all three metrics. On the other hand, for subchallenge 1, for which the goal is to select 60 genes to best approximate the cell locations determined by 84 genes, random gene selection coupled with neighbor-based reweighting achieved almost the same performance as our unsupervised approach, and is only slightly inferior to the supervised approach. This is understandable because of the extensive overlap between the randomly selected genes and the “optimal” gene set. High degree selection achieved somewhat less accurate results than random selection, indicating that some less frequently expressed genes are important determinants of cell locations. For subchallenge 2, our proposed methods outperformed all four baseline approaches in s2, and three out of the four baseline approaches in s1 and s3. Finally, comparing the four baseline approaches suggest that the neighbor-based reweighting component significantly improved s2, but its impacts on the other two metrics are somewhat mixed. Overall, the significant performance gain in subchallenge 3 compared to random gene selection and high degree gene selection supports that the small set of genes we identified are important for predicting cell locations.

**Table 1.** Numerical values of subchallenges scores are given for the ease of comparison with some designed baseline methods.

	SubCh1			SubCh2			SubCh3		
gene selection method	s1	s2	s3	s1	s2	s3	s1	s2	s3
**Group A**									
Our Unsupervised method	0.6610	1.4522	0.6122	0.6552	1.3176	0.6538	**0.6620**	1.0166	**0.7928**
Our Supervised method	0.6730	**1.5463**	0.5937	0.6558	**1.3719**	**0.6731**	0.6534	**1.0994**	0.7807
**Group B**									
Random gene selection	0.6736	1.0638	**0.6289**	0.6113	0.6930	0.6240	0.5362	0.5052	0.7283
Random gene selection + neigh based reweighting	0.6714	1.4043	0.5762	**0.6642**	1.139	0.6619	0.5734	0.6997	0.6964
High degree gene selection	**0.6914**	0.904	0.5860	0.6061	1.0163	0.5969	0.5653	0.6156	0.7159
High degree gene selection + neigh based reweighting	0.6702	1.3241	0.5706	0.6134	1.027	0.5978	0.5593	0.7065	0.6468
Avg of all metrics in group A for each subchallenge		0.9231			0.8879			0.8342	
Avg of all metrics in group B for each subchallenge		0.8137			0.7376			0.6291	

The predictions mentioned above did not involve any additional pre-processing steps, e.g. imputation, on the provided input data. We simply used the binarized and normalized *in situ* hybridization and scRNA-seq data. However, for the sake of completeness we also examined the possible role of "imputation" and using raw data instead of the binarized scRNA-seq data. We tried to impute the dropouts in scRNA-seq data using SAVER ^19^ and netImpute ^20^, but no significant improvement was gained in terms of enhancing our metric scores. On the other hand, although our analysis indicated that using raw data instead of the binarized data can potentially increase the consistency between gene expression pattern similarity and cell proximity in this challenge (according to *M*_1_ and *M*_2_ metrics), we are limited by the fact that the true locations of the cells to be predicted are unknown, and prediction accuracy is at least partially defined by comparing to the “gold standard” location obtained from binarized data. We speculate that anyone using raw data would probably be disadvantaged. It is noteworthy that our method is applicable if one prefers to use raw data instead of binarized data, and our results (data not shown; available as underlying data) indicate that there is benefit of using raw data instead of binarized data.

### Robustness of marker genes

**Figure 1.**
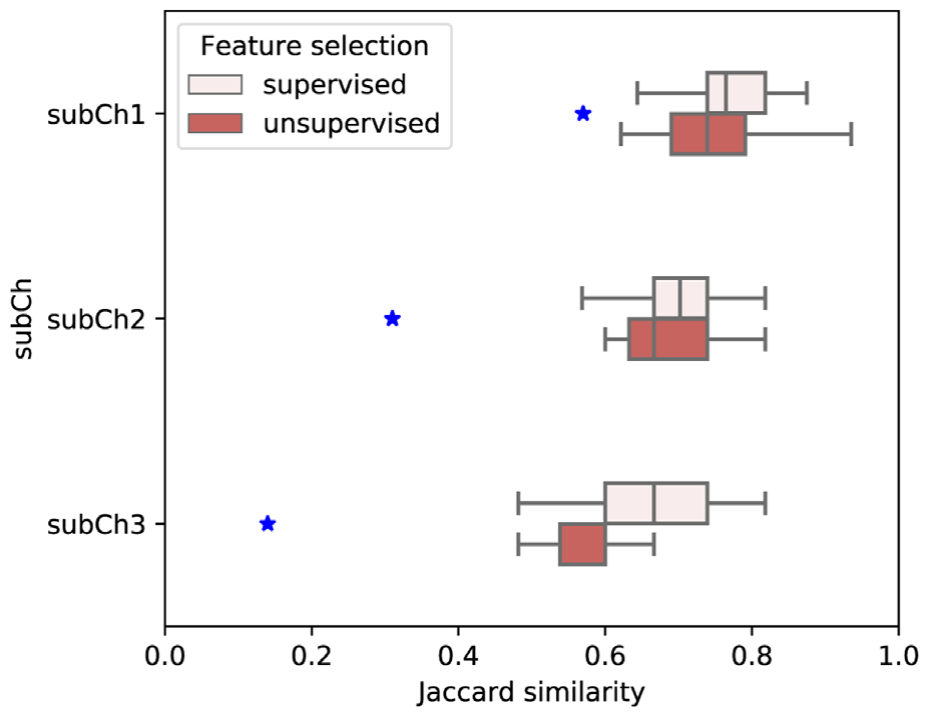
Boxplot shows the Jaccard similarity between the genes selected for each of the 10 CV scheme in all 3 subchallenges. Blue stars represent expected Jaccard similarity.

In the post-challenge phase of the competition the data set was divided into train and test subsets using 10-fold cross-validation in order to further investigate to what degree the set of most informative genes are consistent across different subset of cells selected through the 10 fold CV analysis. The results given in Figure 1 show that the Jaccard similarity between different folds are higher than the expected similarity in all three subchallenges indicating that there in fact exists a consistency in the most-informative genes selected across different folds. Moreover, as the number of genes allowed in a subchallenge decreases (from subchallenge 1 to subchallenge 3) the difference between Jaccard similarity of the most-informative genes and its expected value becomes more and more pronounced.

**Figure 2.**
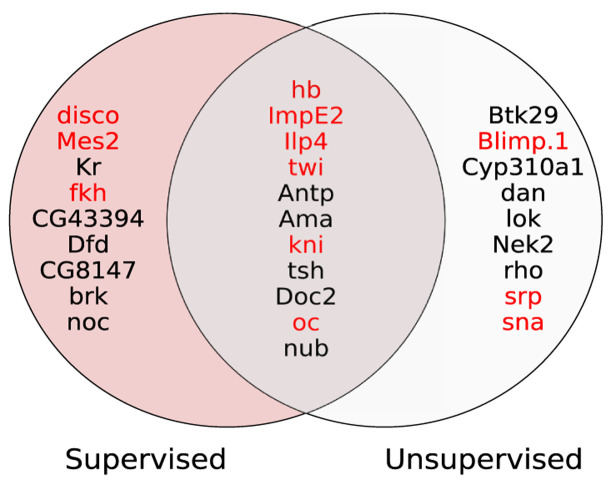
The Venn diagram shows 20 genes selected from supervised and unsupervised methods out of which 11 genes are common for both methods. The 12 genes denoted by red color are the scRNA cluster-specific genes reported in DistMap.

Figure 2 shows the Venn diagram of 20 most informative genes selected from supervised and unsupervised methods. Out of 20 genes selected by each method, there are 11 common genes identified by both methods, which is more than expected (p-value < 0.0005, Fisher’s exact test).

Another interesting observation is that cluster-specific genes (denoted by red color) are prevalent in the set of most informative genes obtained from both supervised and unsupervised methods. This finding highlights our method was in fact able to take advantage of those 12 cluster-specific genes which contain cell location information.

### Recovering gene expression pattern

**Figure 3.**
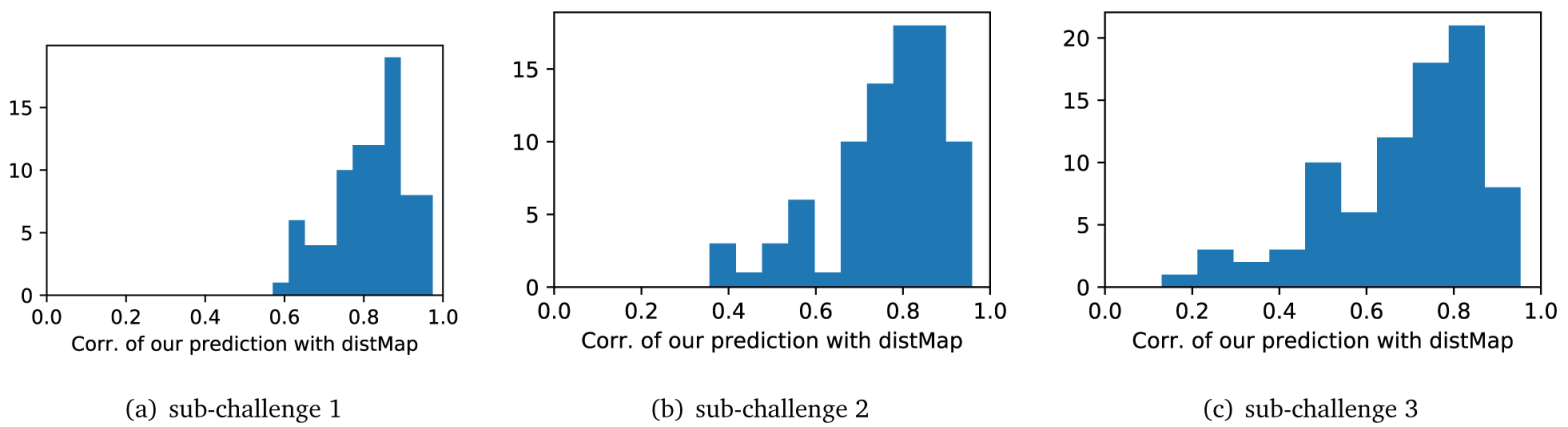
Histogram of correlation between gene patterns predicted by our method and DistMap using 60,40,20 genes.

To virtually reconstruct gene expression patterns, the result of our method (i.e. bin-cell association matrix) was processed based on the methodology of vISH - a tool developed in 16 to derive the expression pattern of each of the 84 genes across the location bins, and compared with the expression patterns obtained by DistMap. Figure 3 shows the distribution of the PCC between DistMap and our results from the three subchallenges. Overall, there is a high correlation among reference patterns (DistMap) and patterns generated by our method. The average correlation in the three sub-challenges are 0.81, 0.76, and 0.68, respectively. In sub-challenge 1, almost all genes have been reliably reconstructed, while for sub-challenge 3, a small number of genes have fairly low reconstruction rate.

**Figure 4.**
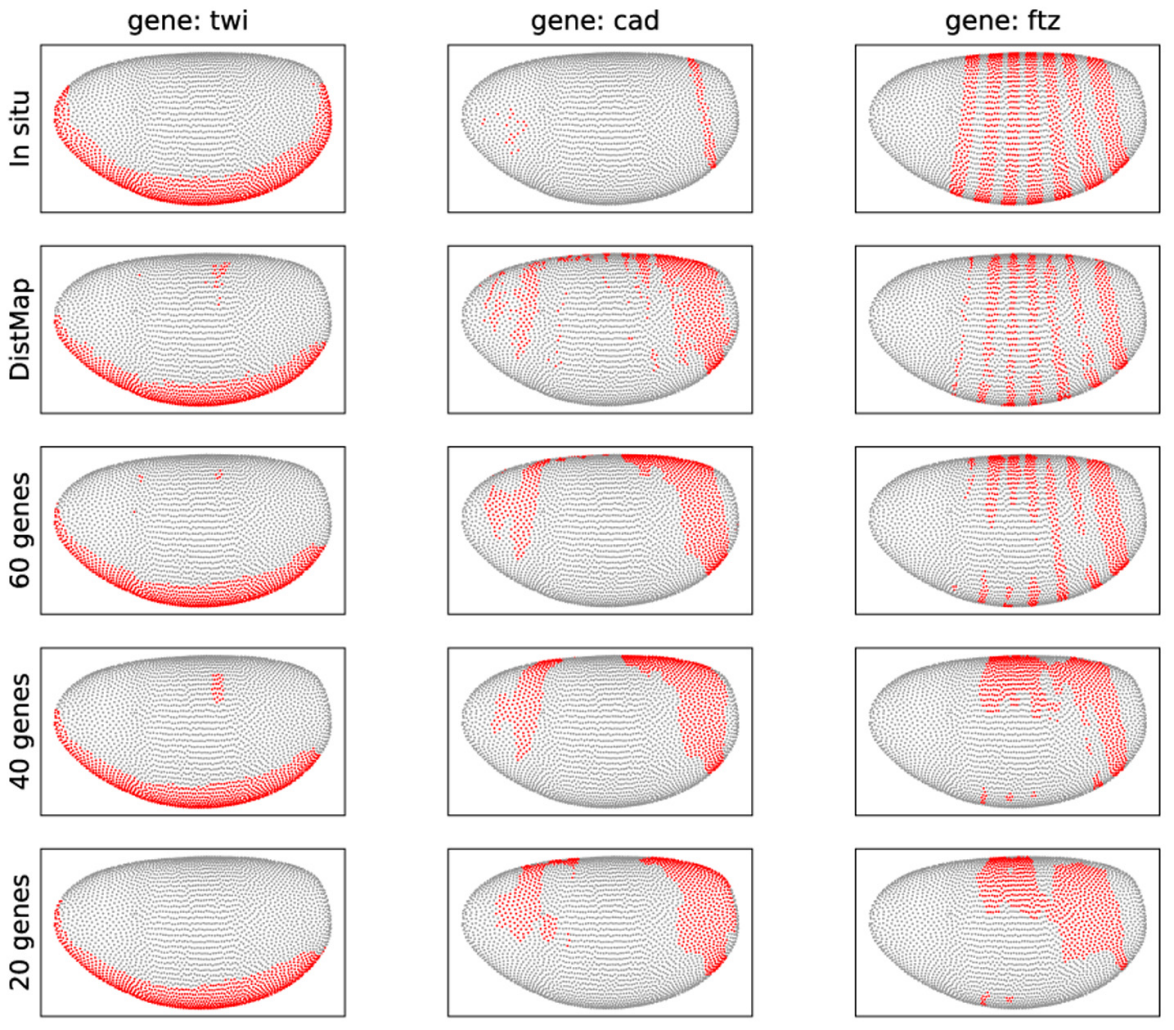
Expression pattern of three sample genes are given for *in situ*, DistMap, and our method using 60,40,20 genes.

Figure 4 shows the reconstructed expression patterns for three genes: *twi*, *cad*, and *ftz*, which play key roles in the regulatory network of early *Drosophila* development. Overall, there is good agreement between our predictions and that of DistMap. In case of *twi*, our method and DistMap both very precisely predicted the *in situ* expression pattern. In fact, *twi* is one the 20 genes selected by both the supervised and unsupervised feature selection methods, due to its distinct expression patterns associated with cell spatial arrangement in the embryo. For *cad*, DistMap and our method with as few as 20 genes predicted very similar expression patterns, where there is a higher expression in the posterior domain, consistent with the current knowledge of cad in embryo development ^21^. On the other hand, the predicted expression patterns seem to be much more diffused than the *in situ* expression pattern, potentially because of the binarization of the *in situ* data, which caused loss of weaker signals. Finally, for *ftz*, while the predicted expression pattern by our method with 60 genes is in general agreement with DistMap and *in situ* data, our method with 40 or 20 genes failed to reconstruct the expression pattern of *ftz* associated with the segmentation of *Drosophila* embryos ^22^. While it is possible that more refined parameters such as a smaller number of neighbor cells may improve the prediction of our method, we believe the striped pattern of *ftz* makes it difficult, if not impossible, for any method that aims at a much reduced number of marker genes for spatial mapping.

## Conclusion

In this work, we proposed a method to identify gene markers for RNAseq-based reconstruction of cell spatial information that were lost during single-cell transcriptomics sequencing of *Drosophila* embryo. The main hypothesis of this study is that the topology of the marker gene expression based cell-cell similarity graph should be consistent with the topology of the cell-cell geometric location map. To test the hypothesis, several metrics were defined based on this biological rationale to capture the consistency between gene expression similarity and cell proximity. A greedy step-wise backward elimination feature selection algorithm was implemented to find a set of most informative genes to optimize these metrics. Next, a Particle Swarm Optimization algorithm was developed to obtain optimal gene weights to construct the cell-location association matrix. Finally, the prediction score of a cell’s location was further improved by considering the expression similarity between neighboring locations. It was shown that our method can successfully identify markers genes capable of predicting cell locations with high accuracy. In addition, it was also demonstrated that our method can recover the spatial expression patterns of most embryo marker genes. Even though the method proposed here was custom designed for this *Drosophila* embryo problem, it has the potential to be readily applied to other organisms as well.

## Data availability

### Underlying data

The challenge datasets can be accessed at https://www.synapse.org/#!Synapse:syn16782375

Challenge documentation, including the detailed description of the Challenge design, overall results, scoring scripts, and the clinical trials data dictionary can be found at: https://www.synapse.org/#!Synapse:syn15665609/wiki/582909

## Software availability

Source code is available from: https://github.com/mary77/scSpatialMapping.git

Archived source code at time of publication: https://doi.org/10.5281/zenodo.3877577 ^18^

License: MIT
