# Supplementary material for: Spatial mapping of single cells in the Drosophila embryo from transcriptomic data based on topological consistency
Source: F1000Res. 2021 Feb 9;9:1014. Originally published 2020 Aug 20. [Version 2] doi: 10.12688/f1000research.24163.2 (PMC7993407; doi:10.12688/f1000research.24163.2)
Supplement: Peer review history

# Peer review: Reviewer response for version 2

**DOI:** 10.5256/f1000research.54404.r79202

**Published:** 2021-03-23

**Authors:** Rui Kuang, Tianci Song

**Affiliations:** 1 Department of Computer Science and Engineering, University of Minnesota, Minneapolis, MN, USA; 2 Department of Computer Science and Engineering, University of Minnesota, Twin Cities, Minneapolis, MN, USA

The authors have addressed all the raised concerns in this revision.

Is the rationale for developing the new method (or application) clearly explained?

Partly

Is the description of the method technically sound?

Yes

Are the conclusions about the method and its performance adequately supported by the findings presented in the article?

Partly

If any results are presented, are all the source data underlying the results available to ensure full reproducibility?

Yes

Are sufficient details provided to allow replication of the method development and its use by others?

Yes

Reviewer Expertise:

Computational Biology, Machine Learning, Biological Network Analysis, Single-cell Genomics, Spatial Genomics

We confirm that we have read this submission and believe that we have an appropriate level of expertise to confirm that it is of an acceptable scientific standard.

# Peer review: Reviewer response for version 2

**DOI:** 10.5256/f1000research.54404.r79203

**Published:** 2021-02-18

**Authors:** Adi Tarca

**Affiliations:** 1 Department of Computer Science, College of Engineering, Wayne State University, Detroit, MI, USA

The authors have address all my concerns but one. This reviewer does not agree with the use of “unsupervised” attribute for method M2. Although this reviewer does not dispute the fact that the method was tested, and that it does not overfit the data, I still believe the method is supervised. To give you a conceptually similar example, lets’ say you want to predict the BMI of a person based on some image features. However instead of fitting your model to the actual BMI values you use a proxy for it (e.g. weight) instead. Of course your model will not perfectly recall the BMI values but this does not mean you did not use information disclosing in part the outcome you want to predict. Back to our problem, the authors may not be using the exact bin locations of the single cells (gold standard) but they are using proxies for them derived from the same or partially the same information used to establish the gold standard. Therefore, I would suggest to remove the term unsupervised when describing this method.

Is the rationale for developing the new method (or application) clearly explained?

Partly

Is the description of the method technically sound?

Yes

Are the conclusions about the method and its performance adequately supported by the findings presented in the article?

Yes

If any results are presented, are all the source data underlying the results available to ensure full reproducibility?

Yes

Are sufficient details provided to allow replication of the method development and its use by others?

Yes

Reviewer Expertise:

Machine learning, genomics, statistics.

I confirm that I have read this submission and believe that I have an appropriate level of expertise to confirm that it is of an acceptable scientific standard.

# Peer review: Reviewer response for version 1

**DOI:** 10.5256/f1000research.26653.r69942

**Published:** 2020-09-18

**Authors:** Adi Tarca

**Affiliations:** 1 Department of Computer Science, College of Engineering, Wayne State University, Detroit, MI, USA

The authors describe their approach to the DREAM Single cell prediction challenge that was designed to find optimal subsets of 20, 40, and 60 genes that would allow prediction of single cells locations in a dme embryo with minimal accuracy loss relative to locations based on 84 genes. The method could be useful in similar applications, yet I have the following comments:

 Major:

1. The “Unsupervised gene selection” section defines two metrics (M1 and M2) to assess the quality a given gene set G of size 20, 40, or 60 out of all 84 genes with in situ data available. The optimal set of genes is identified by a greedy algorithm. However, metric M2 can not be considered unsupervised because it takes the samples from the set where prediction of locations are expected (cells from the scRNA-seq data) and correlates their expression values with profiles of all in situ bins and finds those genes that ensure a maximum correlation. While the authors do not use the information of the in situ cell assigned by DistMap to a given single cell (gold standard), by expecting that there should be some 10 bins with gene expression patterns that correlate with the profile of each single cell, they are implicitly creating their own gold standard. Therefore, not only M2 should be stated as supervised instead of unsupervised, the authors should add as a limitation in the discussion the fact that they have used in situ data from all 84 genes in selecting the best subsets of 20, 40, 60 which was against the DREAM challenge rules.
2. The PSO algorithm to set the gene weights optimization seems to be computationally intensive. Can the authors provide some sense of computation time involved?

Minor: 

- The authors state, “We used a standard backward elimination algorithm to identify a subset of genes *G *with the minimal sum of M1 and M2”. I believe they meant identifying the subset of genes G with a minimal loss (decrease) in M1+M2, since you want the sum to remain as high as possible. So in the end this is a maximization not minimization problem.

Is the rationale for developing the new method (or application) clearly explained?

Partly

Is the description of the method technically sound?

Yes

Are the conclusions about the method and its performance adequately supported by the findings presented in the article?

Yes

If any results are presented, are all the source data underlying the results available to ensure full reproducibility?

Yes

Are sufficient details provided to allow replication of the method development and its use by others?

Yes

Reviewer Expertise:

Machine learning, genomics, statistics.

I confirm that I have read this submission and believe that I have an appropriate level of expertise to confirm that it is of an acceptable scientific standard, however I have significant reservations, as outlined above.

# Response

**Published:** 2021-02-01

**Authors:** Maryam Zand

**Affiliations:** University of Texas at San Antonio, USA

We thank the reviewer for the overall positive comments, as well as the detailed critics that have helped to improve the manuscript.

Response to Reviewer2 comments

 The authors describe their approach to the DREAM Single cell prediction challenge that was designed to find optimal subsets of 20, 40, and 60 genes that would allow prediction of single cells locations in a dme embryo with minimal accuracy loss relative to locations based on 84 genes. The method could be useful in similar applications, yet I have the following comments:

 Major:

**(Q1)** The “Unsupervised gene selection” section defines two metrics (M1 and M2) to assess the quality a given gene set G of size 20, 40, or 60 out of all 84 genes with in situ data available. The optimal set of genes is identified by a greedy algorithm. However, metric M2 can not be considered unsupervised because it takes the samples from the set where prediction of locations are expected (cells from the scRNA-seq data) and correlates their expression values with profiles of all in situ bins and finds those genes that ensure a maximum correlation. While the authors do not use the information of the in situ cell assigned by DistMap to a given single cell (gold standard), by expecting that there should be some 10 bins with gene expression patterns that correlate with the profile of each single cell, they are implicitly creating their own gold standard. Therefore, not only M2 should be stated as supervised instead of unsupervised, the authors should add as a limitation in the discussion the fact that they have used in situ data from all 84 genes in selecting the best subsets of 20, 40, 60 which was against the DREAM challenge rules.

**(A1)** Firstly, we agree that the “unsupervised” version of our method does not strictly follow the DREAM challenge rule of not using *in situ* data directly. The reason is that we were not aware of the rule until after our submission. (The rule was posted in the *discussion forum* instead of on the challenge main webpage, and there were a lot of discussions about this issue among other participants as well). This is why we were allowed to submit, “unofficially”, a supervised version after discussing with the DREAM organizers. Despite violating the DREAM challenge rule, we believe the “unsupervised” method we describe here has been evaluated rigorously and the ideas are valuable, based on both post-challenge analysis results, as well as arguments presented below.

 Secondly, we argue that the M2 metric that we proposed is indeed unsupervised instead of supervised learning. In a supervised method, you optimize parameters in order to achieve the minimal deviation from ground truth (or gold standard in this work). Since the gold standard in this work is defined with all 84 genes, when you reduce the number of genes, you expect to gradually increase the gap between the gold standard and the prediction, and the goal is to minimize the gap with K genes. Our proposed metric M2 does not aim to reduce deviation from ground truth. In fact, the values of M2 achieved with the genes that we selected have better M2 values than the initial 84 genes, which potentially indicate that the initial 84 genes are not “optimal”. Another way to look at this is that we do not use the “gold standard” position in determining how good or bad the selected genes are. The definition of M2 relies on *l* *c* *G* , the location bin whose expression profile of gene set G is the most similar to cell c among all location bins. Although initially when G has all 84 genes, *l* *c* *G*  is indeed identical to the gold standard, *l* *c* * , this set of locations moves away from the gold standard when the gene set changes – so you could say that we have a “moving” gold standard if you wish, and the fact is that with 20 genes, *l* *c* *G*  is quite different from *l* *c* * *.*  Furthermore, while we implemented a stepwise backward elimination method to choose G genes from 84 genes, we could have started with any random set of genes or the whole RNAseq dataset (with much longer running time, however). In fact, our analysis results showed that the M2 values on test data can often be better than the M2 values on training data, further supporting that this is not a supervised method.

**(Q2)** The PSO algorithm to set the gene weights optimization seems to be computationally intensive. Can the authors provide some sense of computation time involved?

**(A2)** We have included this information in the revised manuscript:

 “The running time of PSO algorithm is about 15 hours when running on a system with 16 GB of memory. However, the running time could depend on different optimization settings such as parameter alpha and beta.”

 Minor: 

**(Q3)** The authors state, “We used a standard backward elimination algorithm to identify a subset of genes *G *with the minimal sum of M1 and M2”. I believe they meant identifying the subset of genes G with a minimal loss (decrease) in M1+M2, since you want the sum to remain as high as possible. So in the end this is a maximization not minimization problem.

**(A3)** M1 and M2 were defined based on Euclidean distances and our objective was indeed to minimize M1+M2, not to maximize it.

# Peer review: Reviewer response for version 1

**DOI:** 10.5256/f1000research.26653.r69945

**Published:** 2020-09-07

**Authors:** Rui Kuang, Tianci Song

**Affiliations:** 1 Department of Computer Science and Engineering, University of Minnesota, Minneapolis, MN, USA; 2 Department of Computer Science and Engineering, University of Minnesota, Twin Cities, Minneapolis, MN, USA

This manuscript describes the methodology the authors employed to perform well in the DREAM Single cell Transcriptomics Challenge. The theme of the challenge is to select a subset of 84 known marker genes to characterize the spatial gene expression patterns in Drosophila embryo. The team developed methods for unsupervised gene selection, supervised gene selection, gene weighting and neighbor-smoothing based prediction of cell location. Overall, the manuscript provides sufficient detail to reproduce the results and some interpretations. It appears some further improvements can be done as follows:

1. Where it is understood that the team has optimized the hyper-parameter including k, alpha and beta with some fixed values for this dataset. For others to use the same methods on other similar studies, there is no clue how to tune or select the hyper-parameters. Discussion or more experimental results should be provided in this regard.
2. Similarly, it is also a mystery why different strategies of combining the methods are necessary for the three sub-challenges. There is no discussion of how the combinations are selected or optimized.
3. The results in Table 1 are very poorly explained. How to calculate the 14%, 20%, 30% improvement over the three measures is unexplained. Possibly, it is also helpful to explain the metric used to rank different methods in the competition.
4. In Figure 4, why not also plot the original in-situ hybridization and scRNAseq expressions of the three genes for comparison? The use of vISH seems to be unnecessary.
5. In the section "Datasets and pre-processing steps", a subsection explaining the "gold standard" prediction by DistMap should be added. It is confusing in the description of supervised vs unsupervised gene selection, when the DistMap prediction and the nearest locations are used in the measures in equation (2) and (3).

Is the rationale for developing the new method (or application) clearly explained?

Partly

Is the description of the method technically sound?

Yes

Are the conclusions about the method and its performance adequately supported by the findings presented in the article?

Partly

If any results are presented, are all the source data underlying the results available to ensure full reproducibility?

Yes

Are sufficient details provided to allow replication of the method development and its use by others?

Yes

Reviewer Expertise:

Computational Biology, Machine Learning, Biological Network Analysis, Single-cell Genomics, Spatial Genomics

We confirm that we have read this submission and believe that we have an appropriate level of expertise to confirm that it is of an acceptable scientific standard, however we have significant reservations, as outlined above.

# Response

**Published:** 2021-02-01

**Authors:** Maryam Zand

**Affiliations:** University of Texas at San Antonio, USA

We thank the reviewers for the overall positive comments, as well as the detailed critics that have helped to improve the manuscript.

Response to Reviewer1 comments  

 This manuscript describes the methodology the authors employed to perform well in the DREAM Single cell Transcriptomics Challenge. The theme of the challenge is to select a subset of 84 known marker genes to characterize the spatial gene expression patterns in Drosophila embryo. The team developed methods for unsupervised gene selection, supervised gene selection, gene weighting and neighbor-smoothing based prediction of cell location. Overall, the manuscript provides sufficient detail to reproduce the results and some interpretations. It appears some further improvements can be done as follows:

**(Q1)** Where it is understood that the team has optimized the hyper-parameter including k, alpha and beta with some fixed values for this dataset. For others to use the same methods on other similar studies, there is no clue how to tune or select the hyper-parameters. Discussion or more experimental results should be provided in this regard.

**(A1)** We thank the reviewer for bringing up this important point. It should be noted that as the challenge participants were blind to the evaluation metrics at the time of results submission, which made a direct assessment of the performance of the proposed methods inaccessible, our team did not perform extensive parameter tuning. In the revised manuscript we have provided some insights and reasonings behind the selection of hyperparameter values used in our work. This added information may be used as a general guideline for hyperparameter selection for applying the proposed method to other datasets of similar nature. Nonetheless, it should be mentioned that optimum hyperparameters is problem-specific and may be found through ad hoc tests, sensitivity analysis, and detailed investigations. The following has been added in the manuscript.

 “In this work, *k* is set to 10 because the evaluation of the prediction results is based on 10 best locations for each single cell. Also, based on the number of location bins ( *n*), we believe 10 is a reasonable choice for the number of nearest neighbors.”

  “ *alpha *and *beta* are acceleration coefficients, also referred to as trust parameters. *alpha* expresses how much confidence a particle has in itself, while *beta* expresses how much confidence a particle has in its neighboring particles. Particles draw their strength from their cooperative nature and are most effective when *alpha* and *beta* coexist in a good balance. Most applications use *alpha=beta*. Low values for *alpha* and *beta* result in smooth particle trajectories, while high values cause more acceleration, with abrupt movement towards or past good regions. In this study we used *alpha=beta=0.2*, with 200 agents and the maximum number of iterations set to 40. These parameters were manually tuned by observing the values of the fitness function to reach desired search granularity and speed of convergence.”

**(Q2)** Similarly, it is also a mystery why different strategies of combining the methods are necessary for the three sub-challenges. There is no discussion of how the combinations are selected or optimized.

**(A2)** During the challenge period, each team was given a limited number of attempts to test the success of their proposed approach(es) - evaluation results and ranking of all teams were shown in a leaderboard, with no details of the evaluation metrics. It was made clear that different methods could be used for different sub-challenges. Therefore, it was a natural choice for us to try a diverse combination of the main ingredients of the method for different subproblems to get some insights of these ingredients. During the post-challenge stage, further experiments have been designed by the DREAM organizers for additional insights (see ref. [16]). In this manuscript, we have also added a few baseline approaches to test these components (see Table 1). While we could repeat the whole experiments with a common strategy for all three sub-challenges, we thought it would be best for the readers to see the results in a way consistent with our final results submitted to DREAM challenge. Admittedly, we did not use PSO for sub-challenge 2 because of lack of time towards the end of the competition. The rationale of using neighbor weighting in subchallenge 1 and 2 but not in subchallenge 3 is that as subchallenge 3 used substantially fewer genes, the quality of the location prediction may be relatively low and therefore using gene expression information from the *predicted* neighbors could actually degrade the final prediction. The revised manuscript now provides some details of these rationales.

**(Q3)** The results in Table 1 are very poorly explained. How to calculate the 14%, 20%, 30% improvement over the three measures is unexplained. Possibly, it is also helpful to explain the metric used to rank different methods in the competition.

**(A3)** In the revised manuscript we have further explained the results presented in Table1 to make it clearer. The metrics used to rank different methods is now explained in section “ **Evaluation metrics**”. The details of formulation and additional explanation can be found in the reference article [16]. Additional discussion of the evaluation results has also been included in the revised manuscript (see below for your convenience).

 “Table 1 shows the results of our supervised and unsupervised methods on the three subchallenges, evaluated by the three metrics (s1, s2, and s3) proposed by the DREAM challenge organizers. The details of these metrics are discussed in Method Section “DREAM consortium evaluation metrics”. A more detailed analysis of the results and comparison with other top-performing algorithms are presented in 16 and is not repeated here. To obtain some additional insights of our algorithms’ performance, we present here the results of some variations of our proposed methods. Both our supervised and unsupervised methods have two important components, (1) gene selection, and (2) neighbor-weighted cell location prediction, integral to selecting a set of most informative genes and locating cells based on the information buried in cell neighborhood network topology. To understand the importance of these two components, we designed a set of baseline studies incorporating four experiments. In these experiments, the gene selection strategy was replaced by either selecting genes randomly, or selecting genes expressed in the most number of cells (high degree genes). The neighbor-based reweighting component was also removed in two of these experiments.

 The subchallenge scores corresponding to our method (supervised and unsupervised) along with these four baseline studies are listed in Table 1 under the group A and group B, respectively. The method with highest score (s1, s2, s3) in each of the three subchallenges is shown in boldface. It can be seen that the supervised and unsupervised methods (group A) achieved comparable results, and significantly outperformed the baseline approaches (group B) on average, for all three sub-challenges. For subchallenge 3, which is the most difficult task, both of our methods significantly outperformed the baseline approaches in all three metrics. On the other hand, for subchallenge 1, for which the goal is to select 60 genes to best approximate the cell locations determined by 84 genes, random gene selection coupled with neighbor-based reweighting achieved almost the same performance as our unsupervised approach, and is only slightly inferior to the supervised approach. This is understandable because of the extensive overlap between the randomly selected genes and the “optimal” gene set. High degree selection achieved somewhat less accurate results than random selection, indicating that some less frequently expressed genes are important determinants of cell locations. For subchallenge 2, our proposed methods outperformed all four baseline approaches in s2, and three out of the four baseline approaches in s1 and s3. Finally, comparing the four baseline approaches suggest that the neighbor-based reweighting component significantly improved s2, but its impacts on the other two metrics are somewhat mixed. Overall, the significant performance gain in subchallenge 3 compared to random gene selection and high degree gene selection supports that the small set of genes we identified are important for predicting cell locations.”

**(Q4)** In Figure 4, why not also plot the original in-situ hybridization and scRNAseq expressions of the three genes for comparison? The use of vISH seems to be unnecessary.

**(A4)** The original binarized in situ hybridization data is what the first subplot in Figure 4 shows in fact. For other subplots vISH was used because we wanted to compare our results with Distmap which also uses vISH. Moreover, vISH computes the expression pattern for each gene by combining the cell-bin mapping scores with the original normalized gene expression levels.

**(Q5)** In the section "Datasets and pre-processing steps", a subsection explaining the "gold standard" prediction by DistMap should be added. It is confusing in the description of supervised vs unsupervised gene selection, when the DistMap prediction and the nearest locations are used in the measures in equation (2) and (3).

**(A5)** In the revised version we have added “Gold standard cell locations” subsection.

 “For each of the 1297 cells, the Mathews Correlation Coefficients (MCC) is calculated at each of the 3039 location bins between the binarized 84 RNAseq expression values for the 84 driver genes and the binarized in situ expression values for the same 84 genes. The location bin with the maximum value of MCC score is defined as the gold standard location for each cell.”
